# Dr. Charles R. Butler

**Published:** 1915-02

**Authors:** 


DR. CHARLES R. BUTLER.—Resolutions passed by the Cleveland Dental Society.

WHEREAS, Our Heavenly Father in his divine wisdom has seen fit to remove from the sphere of his earthly labors our esteemed friend and brother, Doctor Charles Richard Butler; therefore be it

*Resolved,* That this Society deeply mourns the loss of one of its most cherished charter members, one who ever displayed a kindness of nature and generosity of heart which will always be remembered with the warmest affection.

*Resolved,* That this Society extends its heartfelt sympathy to his bereaved widow, relatives and friends.

*Resolved,* That these resolutions be spread on the minutes of this Society and published in the dental journals, and a copy be forwarded to his widow, Mrs. Jane Eddy Butler, 1685 Van Buren Street, St. Paul, Minn.

H. L. AMBLER,

S. B. DEWEY,

H. F. HARVEY,

J. F. STEPHAN,

W. H. WHITSLAR,

F. M. CASTO,

*Committee.*

